# Impact of genomic testing on urologists' treatment preference in favorable risk prostate cancer: A randomized trial

**DOI:** 10.1002/cam4.6615

**Published:** 2023-10-03

**Authors:** Samuel Carbunaru, Zequn Sun, Cordero McCall, Bernice Ofori, Norma Marshall, Heidy Wang, Michael Abern, Li Liu, Courtney M. P. Hollowell, Roohollah Sharifi, Patricia Vidal, Andre Kajdacsy‐Balla, Marin Sekosan, Karen Ferrer, Shoujin Wu, Marlene Gallegos, Peter H. Gann, Daniel Moreira, Lisa K. Sharp, Carol E. Ferrans, Adam B. Murphy

**Affiliations:** ^1^ Department of Urology New York University Langone School of Medicine New York New York USA; ^2^ Department of Preventive Medicine Northwestern University Chicago Illinois USA; ^3^ Medical College of Wisconsin Medical School Milwaukee Wisconsin USA; ^4^ Department of Urology Northwestern University, Feinberg School of Medicine Chicago Illinois USA; ^5^ Division of Epidemiology and Biostatistics University of Illinois at Chicago Chicago Illinois USA; ^6^ Division of Urology Duke University Durham North Carolina USA; ^7^ Division of Urology Cook County Health Chicago Illinois USA; ^8^ Division of Urology Jesse Brown VA Medical Center Chicago Illinois USA; ^9^ Department of Pathology University of Illinois at Chicago Chicago Illinois USA; ^10^ Department of Pathology Cook County Health and Hospital System Chicago Illinois USA; ^11^ Pathology and Laboratory Services Jesse Brown VA Medical Center Chicago Illinois USA; ^12^ Department of Urology University of Illinois at Chicago Chicago Illinois USA; ^13^ Institute for Health Research and Policy University of Illinois at Chicago Chicago Illinois USA; ^14^ Department of Biobehavioral Nursing Science University of Illinois at Chicago Chicago Illinois USA

**Keywords:** active surveillance, genomics, prostate cancer, treatment decision

## Abstract

**Introduction:**

The Oncotype Dx Genomic Prostate Score (GPS) is a 17‐gene relative expression assay that predicts adverse pathology at prostatectomy. We conducted a novel randomized controlled trial to assess the impact of GPS on urologist's treatment preference for favorable risk prostate cancer (PCa): active surveillance versus active treatment (i.e., prostatectomy/radiation). This is a secondary endpoint from the ENACT trial which recruited from three Chicago hospitals from 2016 to 2019.

**Methods:**

Ten urologists along with men with very low to favorable‐intermediate risk PCa were included in the study. Participants were randomly assigned to standardized counseling with or without GPS assay. The main outcome was urologists' preference for active treatment at Visit 2 by study arm (GPS versus Control). Multivariable best‐fit binary logistic regressions were constructed to identify factors independently associated with urologists' treatment preference.

**Results:**

Two hundred men (70% Black) were randomly assigned to either the Control (96) or GPS arm (104). At Visit 2, urologists' preference for prostatectomy/radiation almost doubled in the GPS arm to 29.3% (29) compared to 14.1% (13) in the Control arm (*p* = 0.01). Randomization to the GPS arm, intermediate NCCN risk level, and lower patient health literacy were predictors for urologists' preference for active treatment.

**Discussion:**

Limitations included sample size and number of urologists. In this study, we found that GPS testing reduced urologists' likelihood to prefer active surveillance.

**Conclusions:**

These findings demonstrate how obtaining prognostic biomarkers that predict negative outcomes before treatment decision‐making might influence urologists' preference for recommending aggressive therapy in men eligible for active surveillance.

## 1 INTRODUCTION

Active Surveillance (AS) has increased dramatically over the last decade as a safe alternative to immediate treatment for patients with favorable risk prostate cancer (PCa). This strategy, however, has not been equally adopted across all racial/ethnic arms, with lower rates seen particularly in Black men.^1^ There are persistent concerns about whether AS is equally safe in this high‐risk group due to several reasons: differences in biological aggressiveness of the cancers, reduced compliance with follow‐up (particularly in safety net hospitals), and potential under‐sampling of tumors given the higher incidence of anterior tumors.^2^, ^3^ National trends in AS implementation suggest that there are multiple factors that influence patients' choice to pursue this strategy, such as socioeconomic status, provider communication/attitudes, and tumor factors.^4^, ^5^ During treatment counseling, the urologist's treatment preferences or recommendations weigh heavily in the patient's treatment choice.^6^

Genomic tests like Decipher Prostate Biopsy Genomic Classifier and the Oncotype DX Genomic Prostate Score® (GPS) are designed to provide orthogonal data beyond standard risk stratification schemas, such as those promulgated by the National Comprehensive Cancer Network (NCCN). Genomic testing refines risk classification, which has been shown to increase uptake in AS.^7^, ^8^, ^9^ However, most of these studies occurred in well‐resourced academic medical centers or enrolled predominantly White participants, so we lack data about their impact in safety net hospitals or in a predominantly Black high‐risk population.^8^, ^10^ Additionally, very few studies have evaluated how genomic testing impacts urologists' treatment preferences at PCa diagnosis using a randomized trial design.

This study, which utilizes data from a randomized controlled trial in a predominantly Black population,^11^ seeks to evaluate the effects of GPS testing on urologists' treatment preference. Furthermore, our secondary objective is to identify additional factors associated with urologists' preference for AS.

## 2 MATERIALS AND METHODS

### 2.1 Study design and participants

From 2016 to 19 we enrolled 200 men into a randomized controlled trial, ENACT (Engaging Newly Diagnosed Men About Cancer Treatment Options) from three sites: the University of Illinois at Chicago (UIC), John H. Stroger Jr Hospital of Cook County (Cook County), and the Jesse Brown VA Medical Center.^11^ Newly diagnosed PCa patients were eligible for AS were invited to enroll if age ≤ 75 years, life expectancy >10 years, Eastern Cooperative Oncology Group 0–2, and NCCN risk group favorable intermediate or below. Due to the lack of a favorable intermediate risk definition, our urologists included four men with cT1c Gleason grade group 2 PCa with a PSA from 10 to 15 ng/mL with a PSA density〈0.15 to accommodate men with very large prostate volumes. The study preceded the introduction of prebiopsy MRIs at our sites, and only three participants obtained an MRI during the study timeframe. All sites followed a 10‐core minimum biopsy protocol, and the majority of participants obtained a standardized 12‐core systematic Transrectal ultrasound guided biopsy.

At their first postbiopsy visit (Visit 1), participants were randomly assigned to Intervention (GPS testing) or Control (no GPS testing), using a block random assignment scheme stratified on trial site and NCCN risk level. Participants would discuss their PCa diagnosis and received brief standardized counseling at this visit to describe the NCCN risk group and guideline‐based treatment options. Baseline demographic and clinical data were collected, including urinary (International Prostate Symptom Score) and sexual function (International Index of Erectile Function), health literacy (Chew's Brief Health Literacy Survey), and psychometric (e.g., Memorial Anxiety Scale for prostate cancer, Hospital Anxiety and Depression scale) indicators. For participants in the Intervention arm, study pathologists selected tumor blocks containing the highest grade and then prioritized the core with the largest tumor volume; tissue sections or blocks were sent to Genomic Health, Inc. for analysis. After 2–3 weeks, participants returned for their second postbiopsy visit (Visit 2), at which time they would go over their GPS report (if they were in the Intervention group), and receive standardized NCCN guideline‐based counseling to decide on a treatment strategy. The GPS report format underwent some changes during the duration of the trial. Version 1 and 2 only differed in the graphical display but presented the same information; versions 3 of the report added estimates of the 10‐year likelihood of metastasis and PCa death. The majority of patients received version 3 (70 participants), 20 received version 2, and only one received the first version.

### 2.2 Participating urologists

A total of 10 urologists participated in the study. At Visit 1, coordinators ascertained the urologists' initial treatment preference prior to evaluating the patient. The only information presented at the time was the patient's name, age, PSA, clinical stage/digital rectal exam results, and their NCCN risk group. Responses were documented using a standardized survey, which allowed urologists to choose from several treatment modalities: AS, radiotherapy, radical prostatectomy, and other. A second survey was obtained prior to the patient's second postbiopsy visit (Visit 2). Urologist would receive the patient's name, NCCN risk group, and GPS report (if randomized to Intervention arm). The urologists' treatment preference recorded at Visit 2 is the primary outcome of this study.

### 2.3 Statistical analysis

In order to assess randomization, *t*‐tests and chi‐squares were used explore the confounding structure within the data. An intention‐to‐treat analysis was performed for the urologists' preference at Visit 2 by using Fisher exact tests. Various logistic regression models were constructed to assess independent associations with urologists' treatment preference at Visit 2. We identified a list of clinical, demographic, socioeconomic, and psychometric covariates, a priori from a nonsystematic PubMed literature search, that were associated with patient treatment choice, higher risk of aggressive prostate cancer, or poor outcomes.

A minimally adjusted binary logistic regression model for active treatment versus AS was developed by adjusting for the urologist's treatment preference at Visit 1. Watchful waiting (*n* = 2) was combined with AS in this analysis. For covariate selection, we performed both backwards selection for variables with *p* < 0.10 and an iterative model building approach using the variables that had univariate associations with treatment preference at Visit 2 with *p* < 0.10. Models with the highest c‐statistic and lowest Akaike Information Criteria (AIC) scores were preferred. Both R and SPSS 28.0 IBM Corp were used for all analyses.

## 3 RESULTS

### 3.1 The trial population

The details of the study population and methods are in included in our primary endpoint paper from 2021.^11^ Briefly, 200 of 317 potentially eligible men were enrolled (see Figure S1 CONSORT diagram) from which 104 were allocated to the GPS arm and 96 to the Control arm. Notably, 70% of men self‐reported as African American or Black. The distribution of NCCN risk group was as following: 40% very low risk, 35% low risk, and 25% favorable intermediate (see Table 1). There was no difference between the Intervention and Control arm in age, race/ethnicity, NCCN risk group, PSA level, Gleason grade, education level, or International Prostate Symptom Score (IPSS), etc. First‐degree family history of PCa was statistically different, which occurred in 31% of Controls compared to 17% of GPS arm (*p* = 0.02).

**TABLE 1:** Clinical and demographic characteristics of the ENACT trial study arms at baseline.

Characteristics^a^	Control (*n* = 96)	GPS arm (*n* = 104)	Total (*n* = 200)	*p* Value
Age, years	63.5 (6.4)	63.8 (6.9)	63.6 (6.6)	0.750
Race/ethnicity				
African American/Black	65 (67.7%)	75 (72.1%)	140 (70.0%)	–
European American/White	16 (16.7%)	17 (16.4%)	33 (16.5%)	–
Hispanic/Latino	15 (15.6%)	10 (9.6%)	25 (12.5%)	–
Asian	0 (0%)	2 (1.9%)	2 (1.0%)	0.371
Clinical Site				
Jesse Brown VA	40 (41.7%)	43 (41.4%)	83 (41.5%)	–
UIC	17 (17.7%)	20 (19.2%)	37 (18.5%)	–
Cook County Health	39 (40.6%)	41 (39.4%)	80 (40.0%)	0.960
Sociodemographic factors				
Less than high school	20 (21.0%)	14 (13.5%)	34 (17.0%)	–
High school	26 (27.0%)	29 (27.9%)	55 (27.5%)	–
Some college	37 (38.5%)	43 (41.4%)	80 (40.0%)	–
Bachelor's degree or above	13 (13.5%)	18 (17.3%)	31 (15.5%)	0.544
Health literacy^b^	8.3 (3.3)	8.9 (2.9)	8.6 (3.1)	0.198
Living alone	37 (38.5%)	37 (35.6%)	74 (37.0%)	0.664
1st Degree family history of PCa	30 (31.4%)	18 (17.3%)	48 (29%)	**0.021**
NCCN risk level				
Very Low	40 (41.7%)	40 (38.5%)	80 (40.0%)	–
Low	34 (35.4%)	36 (34.6%)	70 (35.0%)	–
Favorable Intermediate^c^	22 (22.9%)	28 (26.9%)	50 (25.0%)	0.817
PSA, ng/mL	5.98 (2.54)	5.98 (2.35)	5.98 (2.44)	0.982
Gleason grade group				
GG1 (3 + 3)	82 (85.4%)	80 (76.9%)	162 (81.0%)	–
GG2 (3 + 4)	14 (14.6%)	24 (23.1%)	38 (19.0%)	0.150
Health status				
Charlson Comorbidity Index	2.9 (1.8)	3.0 (1.8)	3.0 (1.8)	0.694
IPSS (urinary function)	9.9 (7.5)	9.4 (6.9)	9.7 (7.2)	0.627
SHIM score (sexual function)	16.0 (7.1)	17.3 (5.6)	16.8 (6.4)	0.175
Urologists' treatment preference at Visit 1				
Radical prostatectomy	10 (10.4%)	15 (14.4%)	25 (12.5%)	–
Radiotherapy	1 (1.1%)	1 (1.0%)	2 (1.0%)	–
AS/WW^d^	85 (88.5%)	88 (84.6%)	173 (86.5%)	0.760

*Note*: Bold indicates statistical significance.

Abbreviations: AS/WW, active surveillance/watchful waiting; ENACT, Engaging Newly Diagnosed Men About Cancer Treatment Options; GG, Gleason grade; IPSS, International Prostate Symptom Score; NCCN, National Comprehensive Cancer Network; PCa, prostate cancer; PSA, prostate‐specific antigen; SD, standard deviation; SHIM, Sexual Health Inventory for Men; UIC, University of Illinois at Chicago; VA, Veterans Affairs Medical Center.

^a^ Mean (SD) and *n* (%) are presented for continuous and categorical variables, respectively.

^b^ Short‐form Brief Health Literacy Score (range 3–15, <10 considered low literacy).

^c^ Definition of favorable intermediate modified from NCCN favorable intermediate as specified in Methods.

^d^ Preference ascertained before Visit 1. AS includes watchful waiting (*n* = 1).

### 3.2 Urologists' treatment preference

The ten urologists that participated in ENACT practiced at academic medical centers classified as safety net hospitals: five urologists were from Cook County Health, two from Jesse Brown VA, and three from University of Illinois at Chicago (UIC). Their ages ranged from 38 to 76 years old, and seven (70%) practice as urologic oncologists (see Table S1).

**FIGURE 1:**
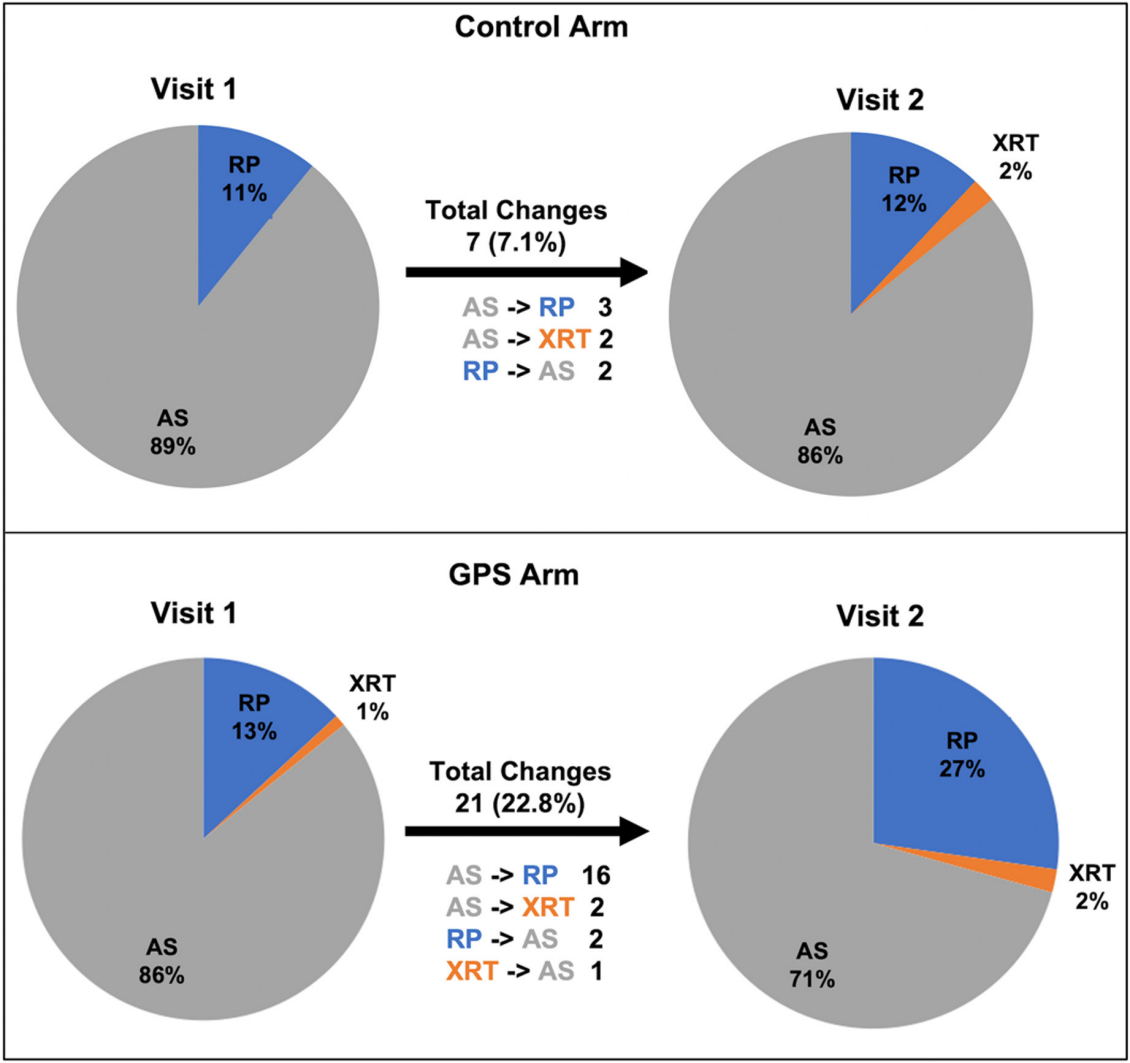
Urologist treatment preference at Visit 1 and Visit 2 stratified by study arm. These pie charts are displaying the distribution of urologists' treatment preference at Visit 1 and 2 from the Control and GPS arms. The changes in treatment preference are shown beneath the arrows along with the number of participants for each treatment change that occurred. The pie chart shows the distribution of urologists' treatment preferences at Visit 1 (left) and at Visit 2 (right) across the three treatment options where active surveillance and watchful waiting are combined. AS, active surveillance/watchful waiting; RP, radical prostatectomy; XRT, radiotherapy.

As seen in Table 2, urologists' preference at Visit 1 for active treatment (radical prostatectomy or radiation) was 11.4% (11) in the Control arm and 15.3% (16) in the GPS arm (*p* = 0.42). Nine participants dropped out after Visit 1, leaving 191 urologists' treatment preferences evaluable. At Visit 2, once GPS scores were available, urologists' preference for active treatment almost doubled in the GPS arm to 29.3% (29) versus to 14.1% (13) in the Control arm (*p* = 0.01). We depict this shift in treatment preference in Figure 1 by study arm.

**TABLE 2:** Association of random assignment to GPS assay with urologists' treatment preference at Visit 2 (primary endpoint).

Initial urologists' treatment preference at Visit 1 by study arm			
	Study arm		*p* = 0.42^a^
Initial urologists' treatment preference	GPS arm *N* (column %)	Control arm *N* (column %)	Total *N*
Active surveillance or watchful waiting (*n* = 3)	88 (84.6%)	85 (88.5%)	173
Radical prostatectomy or radiation	16 (15.4%)	11 (11.5%)	27
Total	104	96	200

Final urologists' treatment preference at Visit 2 by study arm			
	Study arm		*p* = 0.01^a^
Final urologists' treatment preference	GPS arm *N* (column %)	Control arm *N* (column %)	Total *N*
Active surveillance or watchful waiting (*n* = 1)	70 (70.7%)	79 (85.9%)	149
Radical prostatectomy or radiation	29 (29.3%)	13 (14.1%)	42
Total	99	92	191

*Note*: This Chi‐square analysis is the primary outcome which is assessing the association between arm of randomization and urologists' preference at baseline in Visit 1 and after potential exposure to the GPS assay at Visit 2. Treatment preference was assessed immediately before Visit 1 and before Visit 2 to lessen the influence of the participants' discussion. Five participants dropped out in the GPS arm and four dropped out in the Control arm.

Abbreviation: GPS: Genomic Prostate Score.

^a^ Chi‐square tests were performed to compare treatment distributions by study arm.

**FIGURE 2:**
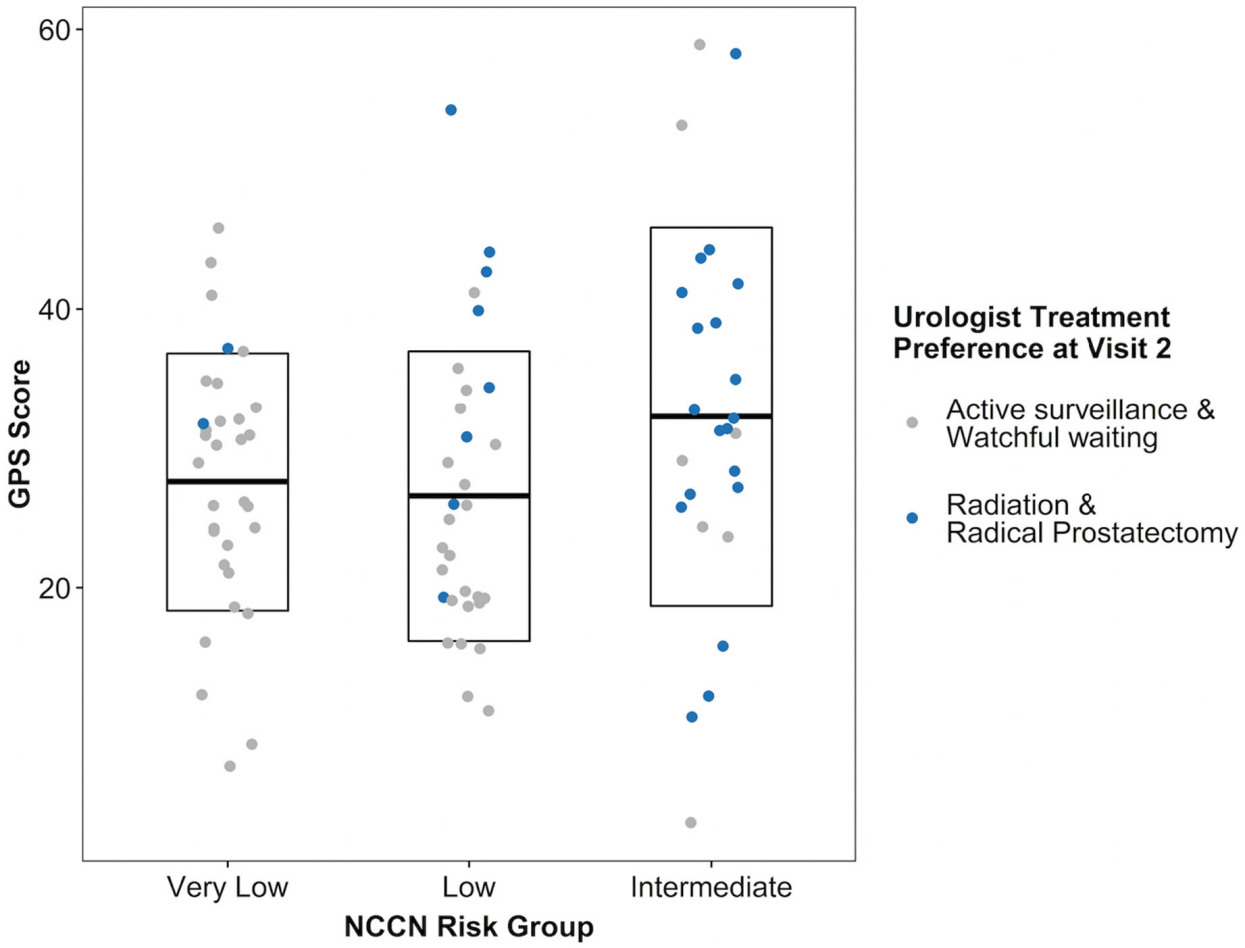
Relationship of GPS to urologists' treatment preference within NCCN risk level: GPS arm only. This is a scatterplot of the GPS scores across the NCCN risk groups with the overlying boxplot showing the median and interquartile range of GPS scores for each risk group. The dots represent the urologists' treatment preferences at Visit 2 which are color coded as blue for active treatment and gray for active surveillance/watchful waiting. The mean GPS for each risk level: very low = 27.4, Low = 27.1, and Favorable intermediate = 32.4. GPS, Genomic Prostate Score; NCCN, National Comprehensive Cancer Network.

Figure 2 shows urologists' treatment preference at Visit 2 for each NCCN risk group based on GPS score. Prostatectomy/radiation was preferred for patients with higher NCCN risk groups, as well as those with higher GPS scores; this is rare in the very low risk group.

In an intention‐to‐treat analysis using an unadjusted binary logistic regression, assignment to GPS was associated with 2.55 higher odds for urologists preferring active treatment (95% CI: 1.25–5.43; *p* = 0.01) relative to men in the Control arm at Visit 2 (see Table 3A). A minimally adjusted model for the urologists' baseline treatment preference at Visit 1 (Table 3A), showed that receiving GPS assay was associated with an increased odds of choosing prostatectomy/radiation by 3.22 (95% CI: 1.38–8.21, *p* = 0.01).

**TABLE 3:** Minimally‐adjusted (A) and fully‐adjusted (B) regressions for urologists' treatment preference for prostatectomy/radiotherapy at Visit 2 (*n* = 191).

Covariates	Categories	Univariable associations odds ratio (95% CI, *p* Value)	Multivariable associations odds ratio (95% CI, *p* Value)
A			
Study arm	Control arm (ref)	–	–
	GPS arm	**2.55 (1.25–5.43, *p* = 0.012)**	**3.22 (1.38–8.21, *p* = 0.009)**
Urologists' Treatment Preference at Visit 1	Active surveillance/watchful waiting (ref)	–	–
	Prostatectomy/radiation	**19.83 (7.54–59.40, *p* < 0.001)**	**23.01 (8.30–74.08, *p* < 0.001)**
B			
Study arm	Control arm (ref)	–	–
	GPS arm	**2.52 (1.24–5.36, *p* = 0.01)**	**3.25 (1.25–9.38, *p* = 0.02)**
NCCN risk group	Very low/low (ref)	–	–
	Favorable intermediate	**10.39 (4.86–23.14, *p* < 0.001**	**7.04 (2.77–18.73, *p* < 0.001)**
Education	No High school degree (ref)	–	–
	High school completed	**4.63 (1.31–29.46, *p* = 0.04)**	8.69 (1.18–123.35, *p* = 0.06)
Health literacy, range 3–15	Continuous	0.90 (0.77–1.06, *p* = 0.20)	**0.78 (0.62–0.99, *p* = 0.044)**
Site	Jesse Brown VA (ref)	–	–
	Stroger	0.45 (0.19–1.00, *p* = 0.06)	0.51 (0.16–1.54, *p* = 0.24)
	UIC	1.26 (0.52–2.96, *p* = 0.60)	0.55 (0.12–2.04, *p* = 0.39)
MAX‐PC score at Visit 1, range 0–54	–	**1.04 (1.01–1.07, *p* = 0.013)**	1.01 (0.96–1.05, *p* = 0.76)
Urologists' treatment preference at Visit 1	Active surveillance (ref)	–	–
	Prostatectomy/radiation	**23.79 (8.64–77.64, *p* < 0.001)**	**20.03 (5.59–88.01, *p* < 0.001)**

*Note*: We include the univariate associations for each covariate that is included in the multivariable models using odds ratios and 95% confidence intervals. Chew's brief screening survey of health literacy is scored from 3 to 15 with scores <10 indicating low health literacy.

The bolded values highlight covariates with associations with urologists' treatment preference with a *p* value < 0.05.

Abbreviations: CI, Confidence interval; *GPS, Genomic Prostate Score (0–100); MAX‐PC, Memorial Anxiety Scale for Prostate Cancer (0–54, >27 is clinically anxious); NCCN, National Comprehensive Cancer Network risk group as a binary variable; ref, reference category; UIC, University of Illinois at Chicago; VA, Veterans Affairs Medical Center.

We include the best‐fit multivariable model in Table 3B. Urologists were 3.25 times more likely to prefer prostatectomy/radiation for men in the GPS arm compared to the Control arm (95% CI: 1.25–9.38, *p* = 0.02). The urologists' baseline preference at Visit 1 was the strongest predictor of urologists' preference at Visit 2 (OR 20.03; 95% CI: 5.59–88.0, *p* < 0.001). Relative to men in the low and very low NCCN risk category, urologists were almost seven times more likely to prefer prostatectomy/radiation for men with favorable intermediate risk PCa. By contrast, increasing patient health literacy was associated with higher likelihood of choosing AS (OR: 0.78, 95% CI: 0.62–0.99, *p* = 0.04). High school completion had a borderline association with preference for prostatectomy/radiation. Site and patient's PCa related anxiety (MAX‐PC scale) were not statistically significant, but the Akaike Information Criteria (a measure of prediction error) and C‐statistics suggested improved model accuracy.

**FIGURE 3:**
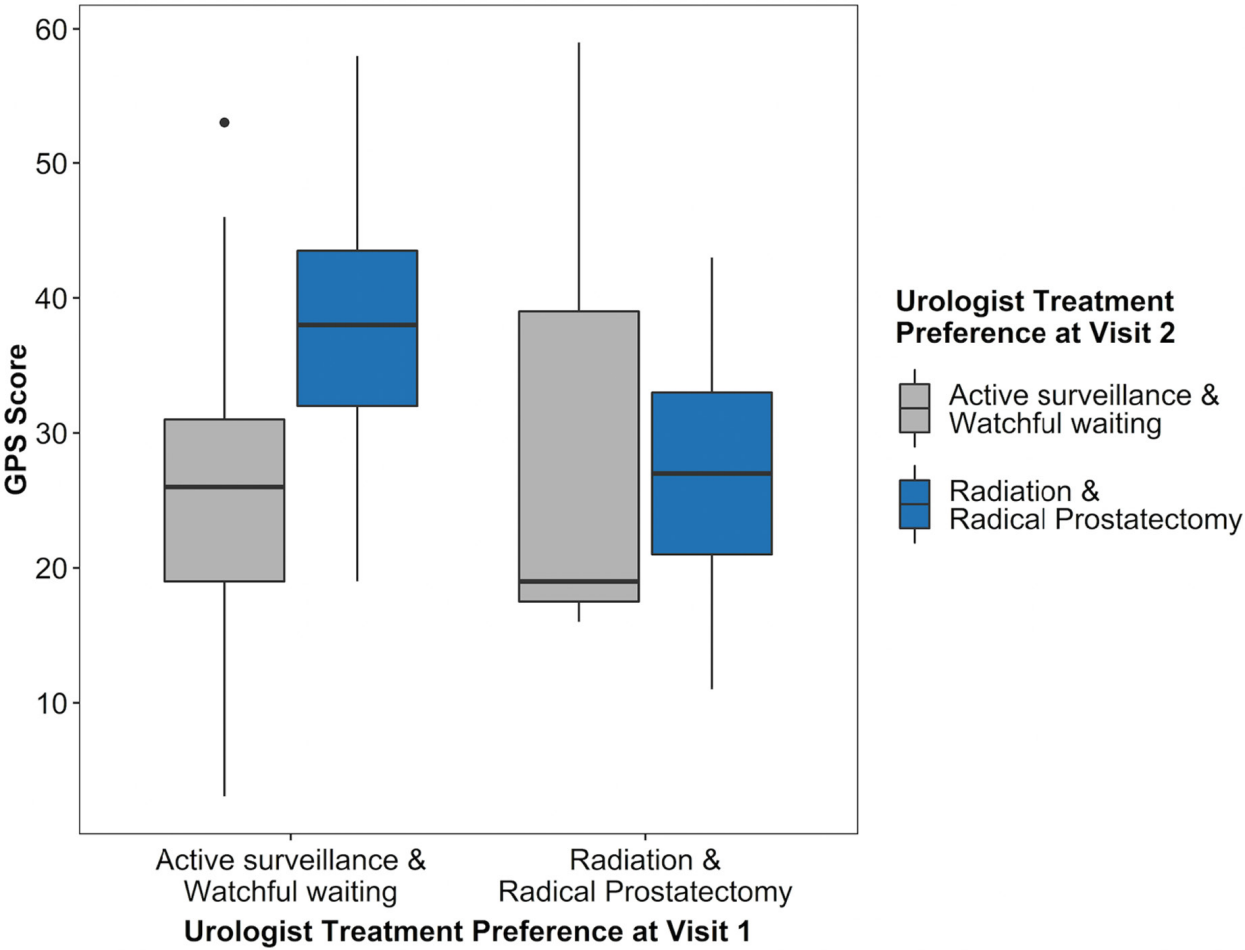
Boxplot of GPS scores by urologists' initial treatment preference stratified by urologist treatment preference at Visit 2 (*n* = 91, GPS Arm only). This is a boxplot of the GPS scores for men in the GPS arm stratified by urologists' treatment preference at Visit 1 and further stratified by urologists' treatment preference at Visit 2. This is a visual representation of the multiplicative interaction in Table S2 and shows that higher GPS scores were associated with providers changing their preference from active surveillance to radical prostatectomy or radiotherapy (i.e., active treatment).

Breaking randomization, we constructed a third model within the GPS arm (*n* = 91) to assess the independent effect of the GPS score and the report's change in NCCN risk group on urologists' treatment preference. On univariate modeling, GPS and GPS‐induced change in NCCN group both were statistically associated with urologists' preference for prostatectomy/radiation. There was a borderline association for a GPS‐induced increase in NCCN risk group having 7.5 times higher odds (95% CI: 1.00–81.13, *p* = 0.06; see Table S2) for urologists preferring active treatment. A significant interaction with GPS score and urologists' treatment preference at Visit 1 was noted (see Figure 3). Among the men whose urologists initially preferred AS, the mean [SD] GPS scores were significantly higher for those whose urologists changed their treatment preference to active treatment at Visit 2 relative to men whose urologists continued to prefer AS (mean 37.8 [9.3], vs. 25.9 [9.7], *p* < 0.001). The mean GPS scores were not different between men whose urologists initially chose active treatment at Visit 1 and continued with active treatment compared to those whose urologist changed their preference to AS.

Finally, a fourth model was constructed within the GPS Intervention group (*n* = 91) to assess the independent effect of the relative position of their patient's GPS score in the GPS distribution for the patient's baseline NCCN risk group, which is a prominent feature of the GPS report (see Table S3). Quartiles 2 and 3 were combined and coded as average risk, while quartile 1 was coded as a lower risk GPS score and quartile 4 was coded as higher risk. On the multivariable model, having a GPS score in quartile 1 decreased the odds of urologists preferring immediate treatment by 20 times (OR 0.05, *p* = 0.03). Patient race, individual provider, nor site were independent predictors of baseline ot final treatment proference on chi‐square tests.

## 4 DISCUSSION

Compared to previous retrospective studies, this study provides prospective level evidence through a randomized controlled trial to examine the clinical utility of genomic assays on provider treatment preference for PCa. Adding to its uniqueness, it also takes place in a safety net setting with a large percentage of Black men, who underutilize AS. Given the disparities that exist in rates of AS in Black men, and the inclusion of genomic testing into PCa treatment guidelines, it is important to understand how GPS testing might influence counseling by providers.

In this study, we show how in this largely Black cohort, urologists' preference for prostatectomy/radiation almost doubled for patients who received GPS testing relative to patients who did not. In multivariable models, obtaining GPS testing was associated with an increased preference for prostatectomy/radiation, even after accounting for urologists' initial preference prior to genomic testing. Patients with favorable intermediate risk level and low health literacy were also significant predictors for urologists' preference for prostatectomy/radiation at Visit 2. Our prior publication showed that GPS also modestly increased patient's choice for prostatectomy/radiation, especially among men with low health literacy; it is possible that urologists' preferences drove that association.^11^

In the GPS arm subanalysis of GPS‐induced shifts in NCCN risk group, we found an interesting interaction between GPS scores and urologists' initial treatment preference. Using boxplots, we found that higher GPS scores seemed to shift urologists' preferences from AS to active treatment, but lower scores did not frequently shift preferences from active treatment back to AS. In fact, the GPS scores of men whose urologists initially preferred active treatment but changed to AS (*n* = 5) were similar and slightly higher than the GPS scores of men whose urologists continued to prefer active treatment (*n* = 19, *p* = 0.62). However, in a supplemental analysis of the GPS arm subanalysis, we found that GPS scores in the lowest quartile strongly increased urologists' preference for AS relative to quartiles 2–4. The effect of having a GPS score in the lowest quartile was nearly equal in magnitude (OR 0.05 vs. 22.4) as the urologists' baseline preference for active treatment, but in opposite directions. There were times where the urologists' treatment preferences did not align with NCCN recommendations for that patient's risk group given the NCCN's strong preference for AS in very low risk men. While we attempted to adjust for known predictors of treatment preference, there were likely unmeasured factors that contributed to provider decisions, such as perceived patient compliance or trust.

Growing evidence highlights potential racial disparities that exist when recommending AS for PCa. For instance, a recent study of more than 51,000 veterans with low‐risk and intermediate‐risk PCa found that after adjusting for several clinical and demographic factors, Black veterans were less likely to receive AS.^12^ Similar results were reported in a study that used data from SEER‐Medicare, showing lower rates of AS in Black patients.^1^ Some argue that the decreased rate of AS in this population is due to concerns of poorer AS compliance and more aggressive disease seen in Black men that might be associated with disease progression while pursuing conservative management.^13^, ^14^ Yet, this conclusion is controversial since some studies have not found race to be a predictor of disease progression.^15^ There are still, however, persistent concerns of recommending AS to Black patients that exist within the urological community; in our analyses, however, Black race was not associated with urologist treatment preference.^16^

Within the last decade, genomic testing has gained popularity as an objective measure to assist patients and providers in treatment‐related decision‐making. The Oncotype DX GPS assay is a validated predictor of adverse pathology in both White and Black men.^7^ This test provides greater confidence in the safety of AS for urologists and a large percentage of providers find it useful when counseling patients.^17^ Several studies have noted an increase in urologists' recommendation for AS after GPS testing, with a 24% increase noted in a prospective study by Badani et al., and 22% increase in a retrospective study by Dall'Era et al.^17^, ^18^ Both of these studies were relatively small and had a largely White patient population. In our largely Black ENACT cohort, we found an opposite trend where GPS testing reduced both urologists' and patients' likelihood to choose AS.^11^ Given the similarity of the GPS distributions in our study versus others, this may be due to inherently lower risk tolerance among urologists in safety net hospitals for adverse pathology risk; though urologists in both arms exceeded national trends for preference of AS and were similar to AS utilization rates in Sweden.^19^, ^20^, ^21^ Based on the disproportionate number of Black patients who are not being recommended AS relative to their White counterparts, it is particularly important to assess how genomic testing influences urologists' treatment recommendations in this population.

On the contrary, recruitment centers, such as those included in this study, are known for having high rates of high‐risk PCa.^22^ Only 317 (24.1%) of 1315 men with newly diagnosed PCa had favorable risk PCa in our centers, which is evidence of a high‐risk population.^8^, ^10^, ^23^, ^24^, ^25^, ^26^ Moreover, in safety‐net settings, compliance concerns, and poor access to advanced imaging, biopsy techniques, and fellowship‐trained radiologists make AS less rigorous than in larger cohorts.^3^, ^27^, ^28^ The GPS score may represent an objective data point to further limit AS to those least likely to harbor adverse pathology at prostatectomy. Long‐term AS compliance and the degree of overtreatment can only be established with long term follow up in larger cohorts of AS‐eligible men.

Although this was a secondary analysis from a randomized controlled trial, there are several limitations that should be noted. The number of urologists that participated in the study was limited given that we wanted counseling sessions to be as consistent as possible and increasing the pool size might have influenced the standardization of the protocol. The study was a multicentered trial conducted at three different Chicago‐based hospitals, yet all technically considered safety‐net hospitals, which serves a specific patient population. Forty percent of our patients harbored very low‐risk PCa, which might limit the generalizability of the results. Given the small number of events studied, we may have over‐fit the models given the wide 95% confidence intervals. Additionally, the GPS result format changed twice during the trial period, with the most recent version including 10‐year probabilities of death and metastasis. The majority of patients (76%) received the latest version, V3. Ten patients who were assigned to the GPS arm had insufficient tumor samples, so they were unable to process the test. An as‐treated analysis excluding these men in the cohort did not significantly change results from the study.

## 5 CONCLUSION

In this randomized controlled trial, we report how urologists' treatment preference for prostatectomy/radiation increased in men who received GPS score. These findings demonstrate how obtaining prognostic biomarkers at the time of diagnosis might influence urologists' preference for recommending immediate therapy, especially in high‐risk populations.

## AUTHOR CONTRIBUTIONS

**Samuel Carbunaru:** Formal analysis (equal); visualization (equal); writing – original draft (equal); writing – review and editing (equal). **Zequn Sun:** Formal analysis (equal); methodology (equal); software (equal); validation (equal); visualization (equal). **Cordero McCall:** Writing – original draft (equal); writing – review and editing (equal). **Bernice Ofori:** Data curation (equal); formal analysis (supporting); project administration (equal); resources (equal); writing – review and editing (equal). **Norma Marshall:** Data curation (supporting); formal analysis (supporting); writing – review and editing (equal). **Heidy Wang:** Conceptualization (equal); data curation (equal); formal analysis (supporting); methodology (equal). **Michael Abern:** Conceptualization (equal); data curation (equal); funding acquisition (equal); methodology (equal); writing – review and editing (supporting). **Li Liu:** Conceptualization (equal); data curation (equal); formal analysis (supporting); methodology (equal); project administration (equal); software (supporting); validation (supporting); visualization (supporting); writing – review and editing (equal). **Courtney MP Hollowell:** Conceptualization (equal); data curation (supporting); funding acquisition (equal); project administration (equal); resources (equal); writing – review and editing (equal). **Roohollah Sharifi:** Conceptualization (equal); data curation (supporting); methodology (equal); project administration (equal); writing – review and editing (equal). **Patricia Vidal:** Writing – review and editing (equal). **Andre Kajdacsy‐Balla:** Conceptualization (equal); data curation (supporting); writing – review and editing (equal). **Marin Sekosan:** Data curation (supporting); project administration (equal); writing – review and editing (equal). **Karen Ferrer:** Data curation (supporting); project administration (equal); writing – review and editing (equal). **Shoujin Wu:** Data curation (supporting); project administration (equal); writing – review and editing (equal). **Marlene Gallegos:** Data curation (supporting); writing – review and editing (equal). **Peter Gann:** Conceptualization (equal); data curation (equal); formal analysis (equal); funding acquisition (equal); investigation (equal); methodology (equal); project administration (equal); resources (equal); software (equal); supervision (equal); validation (equal); visualization (equal); writing – review and editing (equal). **Daniel M. Moreira:** Data curation (supporting); resources (equal); writing – review and editing (equal). **Lisa K. Sharp:** Data curation (equal); formal analysis (equal); methodology (equal); supervision (equal); writing – review and editing (equal). **Carol E. Ferrans:** Conceptualization (equal); data curation (equal); formal analysis (equal); funding acquisition (equal); investigation (equal); methodology (equal); project administration (equal); resources (equal); software (equal); supervision (equal); validation (equal); visualization (equal); writing – review and editing (equal). **Adam Murphy:** Conceptualization (equal); data curation (equal); formal analysis (equal); funding acquisition (equal); investigation (equal); methodology (equal); project administration (equal); resources (equal); software (equal); supervision (equal); validation (equal); visualization (equal); writing – original draft (equal); writing – review and editing (equal).

## CONFLICT OF INTEREST STATEMENT

The authors certify that all conflicts of interest, including specific financial interests and relationships and affiliations relevant to the subject matter or materials discussed in the manuscript (e.g., employment/ affiliation, grants or funding, consultancies, honoraria, stock ownership or options, expert testimony, royalties, or patents filed, received, or pending), are the following: Dr. Murphy was a paid consultant for Exact Sciences, the prior manufacturer of the Oncotype DX GPS assay, to aid in reformatting their assay report. This was after the study was completed with enrollment and before drafting this manuscript.

## ETHICS STATEMENT

The authors confirm that ethical approval was sought from all relevant Institutional Review Board (IRB) or Ethics Committee prior to commencing this study.

## INFORMED CONSENT STATEMENT

The authors confirm that written informed consent was obtained from all participants of this study.

## Supporting information


Appendix S1

## Data Availability

The data that support the findings of this study are available from the corresponding author upon reasonable request.
