# Supplementary material for: Impact of genomic testing on urologists' treatment preference in favorable risk prostate cancer: A randomized trial
Source: Cancer Med. 2023 Oct 3;12(19):19690–700. doi: 10.1002/cam4.6615 (PMC10587942; doi:10.1002/cam4.6615)
Supplement: Supplementary file 1 — Appendix S1 [file CAM4-12-19690-s001.docx]

**Supplemental Table 1.** Selected Characteristics of the Participating Urologists

| **Urologists’ Demographic Characteristics** | **N (%)** |
| --- | --- |
| **Total Number of Participating Urologists** | 10 (100%) |
| Urologic Oncologist* | 7 (70%) |
| Urologist/other urology subspeciality | 3 (30%) |
| **Number of Urologists by Recruitment Site** | 10 (100%) |
| Jesse Brown VA(recruited 83 patients) | 2 (20%) |
| UIC (recruited 37 patients) | 3 (30%) |
| Cook County (recruited 80 patients) | 5 (50%) |
| **Urologists Racial/Ethnic Characteristics** | 10 (100%) |
| White/Middle Eastern/European American | 6 (60% |
| African American | 2 (20%) |
| Hispanic/Latinx | 2 (20%) |
| **Continuous Characteristics** | **Median (IQR)** |
| Urologists’ Age at Study Initiation, years | 45.0 (37.5-58.2) |
| Time in Practice, years | 11.0 (5.0-25.8) |
| Number of patients enrolled per urologist | 8.0 (3.0-33.5) |

**Reference:** *Three of the urologic oncologists were fellowship trained and four clinically practice as urologic oncologists.

Abbreviations: UIC, University of Illinois at Chicago; VA, Veterans Affairs Medical Center

**Supplemental Table 2.** Fully-adjusted Regressions for Urologists’ Treatment Preference for Radical Prostatectomy/Radiotherapy at Visit 2: GPS arm only (n=91)

| **Covariates** | **Categories** | **Univariate Associations Odds Ratio (95% CI, p-value)** | **Multivariable**  **Associations**  **Odds Ratio (95% CI, p-value)** |
| --- | --- | --- | --- |
| GPS Score*  range 3-59 | - | **1.14 (1.07-1.24, p <0.001)** | 1.07 (0.98-1.19, p =0.17) |
| GPS-induced change in NCCN risk group** | No change in NCCN (ref) | - | - |
|  | Decrease in NCCN group | 2.82 (0.58-13.90, p =0.19) | 0.55  (0.01-12.16, p =0.73) |
|  | Increase in NCCN group | **9.04 (2.91-34.85, p <0.001)** | 7.49  (1.00-81.13, p =0.06) |
| NCCN risk group at baseline | Very Low (ref) | - | - |
|  | Low | **3.60 (1.16-13.55, p =0.04)** | 6.35 (0.93-63.52, p =0.08) |
|  | Favorable Intermediate | **22.09 (7.67-81.02, p<0.001)** | **16.09 (2.59-154.58, p =0.006)** |
| Education | No high school degree (ref) | - | - |
|  | High school completed | **4.63 (1.31-29.46, p=0.04)** | 32.50 (1.29-2072.51, p =0.06) |
| Urologist Treatment Preference at Visit 1 | Active surveillance/ Watchful waiting (ref) | - | - |
|  | Radical Prostatectomy/ Radiotherapy | **23.79 (8.64-77.64, p <0.001)** | **3345.80**  **(21.64-2983944.83, p =0.01)** |
| Health Literacy Score (binary) | 3-9 (ref) | - | - |
|  | 10-15 | **0.47 (0.23-0.97, p =0.04)** | **0.18 (0.03-0.87, p =0.046)** |
| GPS Score X Urologist Treatment Preference at Visit 1 | Multiplicative Interaction | **0.85 (0.75-0.97, p =0.01)** | **0.84 (0.71-0.97, p =0.02)** |

**Reference:** This table displays the *post hoc* exploratory analysis which is a best-fit fully-adjusted binary logistic regression with urologists’ preference for active treatment as the outcome to assess the impact of the shift in NCCN risk groups that the GPS assay reports; this analysis break randomization and only includes men in the GPS arm (n=91). We include the univariate associations for each covariate that is also included in the best-fit multivariable model using odds ratios and 95% confidence intervals. There was a statistical trend for increased active treatment preference for men whose GPS report’s shows an increase in NCCN risk group (p =0.06).

Abbreviations: CI, Confidence interval; *GPS, Genomic Prostate Score (0-100); NCCN, National Comprehensive Cancer Network risk group as a binary variable; ref, reference category. Akaike Information Criteria (AIC): 72.1, c-statistic: 0.934.

**Supplemental Table 3.** Unadjusted and Fully-adjusted Regressions for Urologists’ Treatment Preference for Active Treatment at Visit 2: GPS arm only (n=91)

| **Covariates** | **Categories** | **Univariate Association Odds Ratio (95% CI, p-value)** | **Multivariable Association Odds Ratio (95% CI, p-value)** |
| --- | --- | --- | --- |
| GPS Quartile | GPS score in  Quartile 2 & 3 (ref) | - | - |
|  | GPS score in  Quartile 4 | 2.18 (0.76-6.30, p=0.144) | 1.98 (0.43-9.61, p=0.379) |
|  | GPS score in  Quartile 1 | 0.90 (0.18-3.61, p=0.887) | **0.05 (0.01-0.50, p=0.027)** |
| NCCN risk group at baseline | Very low (ref) | - | - |
|  | Low | **3.60 (1.16-13.55, p=0.036)** | 4.36 (0.76-34.54, p=0.113) |
|  | Favorable intermediate | **22.09 (7.67-81.02, p<0.001)** | **50.87 (8.94-472.82, p<0.001)** |
| Education | No high school degree (ref) | - | - |
|  | High school completed | **4.63 (1.31-29.46, p=0.042)** | 17.41 (0.73-1468.28, p=0.138) |
| Urologist’s Treatment Preference at Visit 1 | Active surveillance/ Watchful waiting (ref) | - | - |
|  | Radical Prostatectomy/ Radiotherapy | **23.79 (8.64-77.64, p<0.001)** | **22.41 (2.81-564.17, p=0.013)** |

**Reference:** This table depicts our *post-hoc* best-fit fully-adjusted binary logistic regressions with urologists’ preference for active treatment as the outcome with Quartiles of GPS score as the main independent variable. The GPS score was converted to an ordinal quartile variable based on the distribution of GPS within each NCCN risk level. Quartiles 2 & 3 are the reference group across each NCCN group. This regression only includes the 91 men who received the GPS report and the model assesses the impact of the relative position of the participants’ GPS score on the normal distribution included in the GPS assay report. We include the univariate associations for each covariate that is also included in the best-fit multivariable model using odds ratios and 95% confidence intervals. Akaike Information Criteria (AIC): 70.1, C-statistic: 0.921.

Abbreviations: CI: Confidence interval; GPS, Genomic Prostate Score (0-100); NCCN, National Comprehensive Cancer Network risk group as a 3-level multinomial variable; ref, reference category.

**Supplemental Figure 1.** CONSORT diagram for the ENACT trial

**
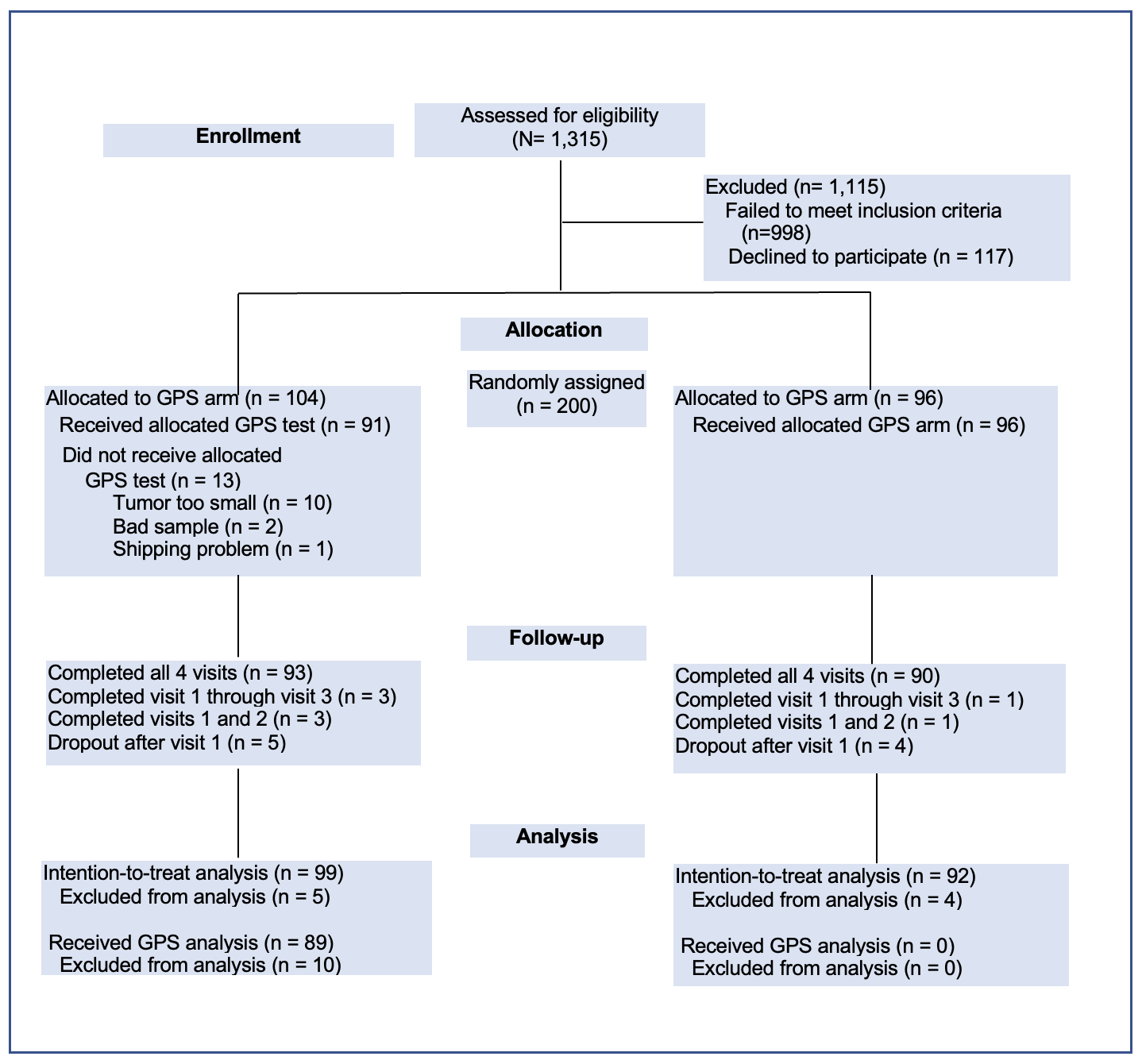
**

**Reference**: CONSORT diagram for the ENACT trial. *Abbreviations: ENACT, Engaging Newly Diagnosed Men About Cancer Treatment Options; GPS, Genomic Prostate Score.
